# Pericyte-Endothelial Interactions in the Retinal Microvasculature

**DOI:** 10.3390/ijms21197413

**Published:** 2020-10-08

**Authors:** Hu Huang

**Affiliations:** Department of Ophthalmology, School of Medicine, Mason Eye Institute, University of Missouri, One Hospital Drive, MA102C, Columbia, MO 65212, USA; huangh1@missouri.edu; Tel.: +1-(573)-882-9899

**Keywords:** blood-retinal barrier, diabetic retinopathy, endothelial cells, exosomes, microvasculature, pericytes, placental growth factor, retina, vessel organoids

## Abstract

Retinal microvasculature is crucial for the visual function of the neural retina. Pericytes and endothelial cells (ECs) are the two main cellular constituents in the retinal microvessels. Formation, maturation, and stabilization of the micro-vasculatures require pericyte-endothelial interactions, which are perturbed in many retinal vascular disorders, such as retinopathy of prematurity, retinal vein occlusion, and diabetic retinopathy. Understanding the cellular and molecular mechanisms of pericyte-endothelial interaction and perturbation can facilitate the design of therapeutic intervention for the prevention and treatment of retinal vascular disorders. Pericyte-endothelial interactions are indispensable for the integrity and functionality of retinal neurovascular unit (NVU), including vascular cells, retinal neurons, and glial cells. The essential autocrine and paracrine signaling pathways, such as Vascular endothelial growth factor (VEGF), Platelet-derived growth factor subunit B (PDGFB), Notch, Angipointein, Norrin, and Transforming growth factor-beta (TGF-β), have been well characterized for the regulation of pericyte-endothelial interactions in the neo-vessel formation processes (vasculogenesis and angiogenesis) during embryonic development. They also play a vital role in stabilizing and remodeling mature vasculature under pathological conditions. Awry signals, aberrant metabolisms, and pathological conditions, such as oxidative stress and inflammation, can disrupt the communication between pericytes and endothelial cells, thereby resulting in the breakdown of the blood-retinal barrier (BRB) and other microangiopathies. The emerging evidence supports extracellular exosomes’ roles in the (mis)communications between the two cell types. This review summarizes the essential knowledge and updates about new advancements in pericyte-EC interaction and communication, emphasizing the retinal microvasculature.

## 1. Introduction

Retinal microvasculature supports the neural retina’s visual function by supplying the retinal cells with nutrients and oxygen and draining the waste out of the tissues. This is critically important because the retina is a metabolically active neural tissue, consuming high levels of oxygen. Retinal microvasculature possesses the specialized features necessary for the maintenance of environmental homeostasis in the retina. One such feature is the blood-retinal barrier (BRB), an analog of the blood-brain barrier (BBB) in the brain, which prevents the free access of blood, especially large molecular weight blood substances, to the delicate neural tissue. As part of the central nervous system (CNS), the retina forms a neurovascular unit (NVU) through the interaction of neurons, vascular cells, and glial cells (astrocytes and Müller) [1]. A retinal NVU is an anatomical structure to retain environmental homeostasis and a function unit to keep retinal cell function. However, various disease and stress conditions, such as ischemia, inflammation, and diabetic retinopathy (DR), can cause the breakdown of BRB, perturb the communication of NVU cells, and disrupt the architecture of retinal microvasculature, thereby exacerbating inflammation, ischemic damage, and pathological process [2,3,4].

Like other microvessels in the CNS and the periphery organs, the retinal microvasculatures contain the two main cellular constituents of endothelial cells (ECs) and pericytes (PCs), sharing the same basement membrane on the blood vessel walls. ECs and PCs communicate via gap junctions at peg-sockets and other paracrine signaling factors, such as growth factor, secreted cytokine, and extracellular exosomes. The two types of cells also interact at adhesion plaques enriched with extracellular matrix (ECM). The communications and interactions are indispensable for the formation, maturation, and stabilization of the CNS vasculatures and their barrier properties during development and adulthood, which are regulated by an array of signaling molecules, such as VEGF, PDGFB, Notch, Angipointein, Norrin, and TGF-β, etc. In contrast, the miscommunications are involved in many pathological and disease conditions, such as ischemic stroke and diabetic retinopathy [5]. Pericyte-endothelial interactions in pathological conditions are disturbed by various factors, such as altered oxygen levels, reactive oxygen species, advanced glycation end products, increased leukocytes adhesion, inflammatory cytokines, and chemokines.

## 2. Endothelial Cell Metabolism and Angiogenesis

Like neurons, ECs rely mainly on glucose for their energy requirements. Glycolysis metabolizes glucose into pyruvate, generating energy (2ATP/glucose) and metabolic intermediates. Pyruvate can enter the TCA cycle (30 ATP/glucose) or convert into lactate, depending on oxygen availability. Metabolic intermediates of the glycolytic pathway can fuel different side metabolic pathways, such as glycogen metabolism, the pentose phosphate pathway, and the hexosamine biosynthesis pathway. Several additional EC metabolic pathways include fatty acid synthesis, glutamine metabolism, and ornithine cycle. Endothelial cell metabolism plays crucial roles in EC functions; thus, its deregulation leads to EC dysfunction and is involved in various vascular diseases, such as cancer, cardiovascular diseases, and diabetes [6]. Targeting EC metabolism can be a strategy to treat vascular diseases. For instance, blockade of 6-Phosphofructo-2-Kinase/Fructose-2, 6-Biphosphatase 3 (PFKFB3), a key regulator of glycolysis in ECs, normalizes tumor vessels with increased pericytes coverages and enhances EC barrier function through decreased VE-cadherin endocytosis [7].

Increasing evidence supports that endothelial cell metabolism plays a critical role in angiogenesis [8]. EC proliferation and migration rely on the energy from glycolysis, the primary source of ATP production in the endothelium. The glycolytic regulator PFKFB3 plays a critical role in the angiogenic growth of ECs, tip cells in particular. Pharmacological inhibition and gene silencing of PFKFB3 impairs EC activities and reduces angiogenesis mediated by VEGF-Notch signaling [9]. In response to the angiogenic stimuli (i.e., VEGF and FGF2), ECs switch their glucose metabolic pathways toward more anaerobic processes: increased glycolysis, glycogen synthesis, and pentose phosphate pathway [10]. These metabolic pathway adaptations play an inherent role in EC proliferation, migration, and angiogenesis.

## 3. Pericytes in the (Retinal) Microvasculature

Pericytes are highly heterogeneous, depending on their residing vascular beds and the pathophysiological microenvironments. Like the vascular smooth muscle cells (VSMCs) in larger blood vessels, pericytes have a mesodermal origin. Neuroectodermal and endocardial ECs and bone marrow (BM) are also the origins of pericytes in different organ systems [11]. The differences in their phenotypic features include varying morphologic shapes, cell coverages (e.g., CNS vs. periphery vasculatures), and their plasticity in response to stimuli both in vivo and in vitro. For example, pericytes express several characteristic molecular markers, such as PDGFR-β, α-SMA, and CD146. However, they are not unique to pericytes and can be expressed by other perivascular and mesenchymal cells [12].

Pericytes are multi-functional cells, attributing to their plastic feature and regenerative potential. They are the critical cellular element of the NVU in the brain and the retina and contribute to the BRB and BBB formation and maintenance. They also participate in the immune and inflammatory response by producing cytokines in response to pathological stimuli and contribute to angiogenesis by modulating endothelial cell proliferation and migration. Pericytes can be trans-differentiated into myoblast cells and mesenchymal stem cells and reprogrammed into neurons and glial cells [11,13,14,15]. The regenerative capacity and plasticity of pericytes allow them to potentially treat the disorders related to vascular dystrophies such as muscular dystrophy, ischemic stroke, and diabetic retinopathy. The close association of pericytes and endothelial cells facilitates their communication through direct contact, ion exchange via gap junction (e.g., Connexin 43) [16], and other paracrine molecules (e.g., Cathepsin D [17] and Sphinogosine 1-phospate [18]). It can be a therapeutic target to treat vascular disorders such as diabetic vascular complications and pathological angiogenesis [12].

**Figure 1:**
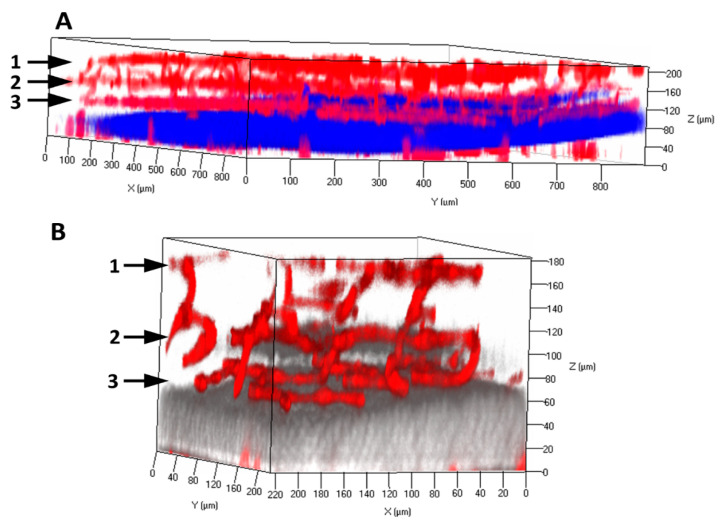
Retinal microvasculature in the adult mouse retina. The retinas were dissected from the adult mice and processed with clarity method. The cleared retinas were performed with the immunofluorescence staining of anti-PECAM1/CD31 primary antibody and Alexa Fluor 594 secondary antibody (red). The three-dimensional (3D) architectures of retinal vasculatures are visualized with low (**A**) and high magnification (**B**). One points to superficial vascular plexus at the nerve fiber layer. Two points to intermediate vascular plexus at the inner plexiform form layer. Three points to deep vascular plexus at the outer plexiform layer. The outer inner nuclear layer and the inner nuclear layer were stained with DAPI. The DAPI signals in Panel B were shown with pseudocolor (grey).

In the differentiated retina of mammals such as mice and humans, the microvasculatures contain the superficial, intermediate, and deep vascular plexuses that localize in the nerve fiber layer (NFL), inner plexiform layer (IPL), and outer plexiform layer (OPL), respectively. Using a clarity method initially described for the mouse brain [19], we cleared the retinal tissues from adult mice. Within the cleared retina, the vascular architectures and networks were explicitly demonstrated with the endothelial marker CD31/PECAM1 staining and confocal imaging (Figure 1). Pericytes density in the retinal microvasculature is relatively high (~1:1 ratio to ECs). It is established that pericytes can restrict EC proliferation and stabilize the blood vessels. However, the exact mechanisms of retinal microvascular pericytes contributing to retinal vascular EC integrity and function are not entirely understood. Retinal microvasculature is particularly sensitive to hypoxia, oxidative, and other stress conditions, such as diabetes mellitus and ischemia stress. Pericytes loss or dysfunction is associated with the symbolic features of diabetic retinopathy: microaneurysm, hemorrhage, acellular capillary, BRB breakdown, and pathological angiogenesis. They result in the loss of pericytes-EC interactions and their mis-communications.

**Figure 2:**
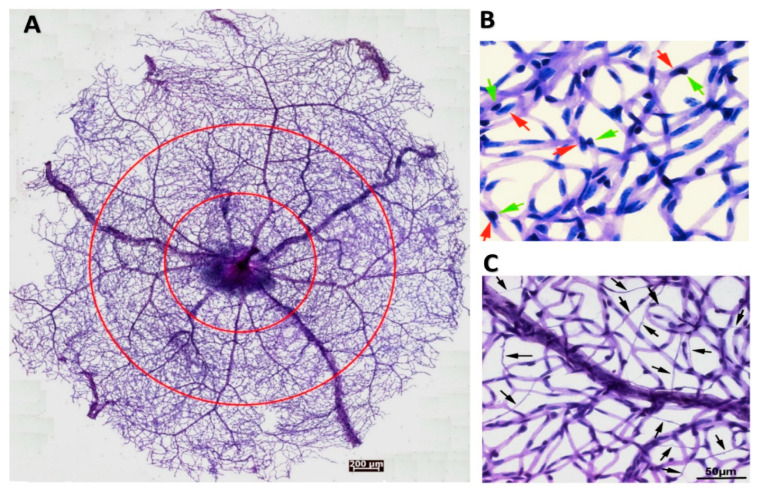
Pericytes-endothelial cell interactions and abnormalities in the retinal microvasculature. (**A**) Overview of an entire retinal microvasculature made from the adult mouse retina with the trypsin digestion method. The circles divided the vascular network into the periphery, middle, and central zones. (**B**) The high magnitude of retinal vasculature indicates the close interactions of pericytes and endothelial cells in the normal blood vessel walls. Red arrows point to the nuclei of endothelial cells. Green arrows point to the nuclei of pericytes. (**C**) The retinal vasculatures are made from the CXCR5 knockout mouse, which was subjected to ischemia-reperfusion injury, leading to a substantial loss of endothelial cells (ECs) and pericytes. Arrows point to the acellular capillary. This image is excerpted from a previous publication with the journal’s permission [22].

Besides the commonly-used confocal imaging and immunofluorescence staining methods, another way to examine and quantitate the retinal vasculatures and their abnormalities is trypsin digestion. This method gently brushes away the neural and other non-vascular tissues from the fixed retina, and the blood vessels are stained with Periodic acid-Schiff reagents [20]. Using this method, we isolated an entire mouse retinal vasculature for quantification based on vessel locations or zones (Figure 2A). Higher magnification images (Figure 2B,C) illustrated the EC-PC interaction and capillary degeneration. Furthermore, we used this method to reveal the aggravated retinal microvascular degeneration caused by ischemia-reperfusion injury (see [21] for the experimental procedures) in the CXCR5 knockout mice compared with the C57BL6/J wild type control [22].

## 4. Establishment of the Pericyte-Endothelial Interactions in CNS Microvasculature

Like the brain microvasculature, retinal micro-vascular formation, growth, and maturation are regulated by a wide range of signaling pathways in a coordinated way during embryogenesis. Excellent reviews discussed these regulatory signaling pathways and their specific roles in the pericyte-endothelial interactions during BBB development and maturation [23,24]. Here, we provide a quick snapshot of several examples and their characterized functions in BBB establishment and stability in brain microvasculature, many of which can be adapted for the BRB in retinal microvasculature. The VEGF-VEGFR2 signaling pathway is essential for the growth and proliferation of endothelial cells during vasculogenesis and angiogenesis processes. Following the neo-vessel formation, the PDGFB-PDGFRβ signaling pathway plays a vital role in pericyte recruitment into the neovessels. Pericyte-endothelial interactions lead to the activation of the latent TGF-beta signaling pathway, which promotes the differentiation and proliferation of ECs and pericyte through its receptors ALK1, ALK5, and endoglin, as well as driving extracellular collagen secretion and CTGF expression by pericytes and astrocytes through Smad 2/3.

Further vascular maturation and stabilization require the angiopoietin1/Tie2 signaling axis, which enhances the EC barrier function through the regulation of VE-cadherin, β-catenin, and the cytoskeleton. Sphingosine-1-phosphate (S1P)/S1P1 receptor signaling axis is vital for pericyte coverage through the regulation of N-Cadherin. Wnt (i.e., Wnt-7a and Wnt7b)/β-catenin signaling axis is essential for the development of CNS vasculature, such as EC barrier properties [25]. Norrin/Frizzled4 signaling is required for retinal vascular development, such as EC proliferation and arterial-venous (A-V) segregation, and also plays a vital role in the maintenance of BRB/BBB integrity and function [26]. Moreover, neuronal guidance cues, such as semaphoring3A (Sem3A) and ephrins, are also important regulators in the neo-vessel formation, particularly the out-growth of the tip cells in filopodia [27,28]. In the retina, ganglion cells are important sources of these guiding molecules and regulate retinal vascular development [29].

## 5. Signaling Pathways Critical in the Mature Microvasculature

Not only are many signaling pathways essential in controlling the angiogenesis during embryonic development, but they also play critical roles in the stabilization and remodeling of the mature vasculatures under pathophysiological conditions. For instance, photoreceptor cells’ signals significantly contribute to DR’s early vascular abnormalities caused by oxidative stress and inflammation [30]. Here, we summarize more information about the three signaling pathways that are critical for the stabilization of the mature vasculatures.

### 5.1. TGF-β-TGFBR Signaling Pathway

**Figure 3:**
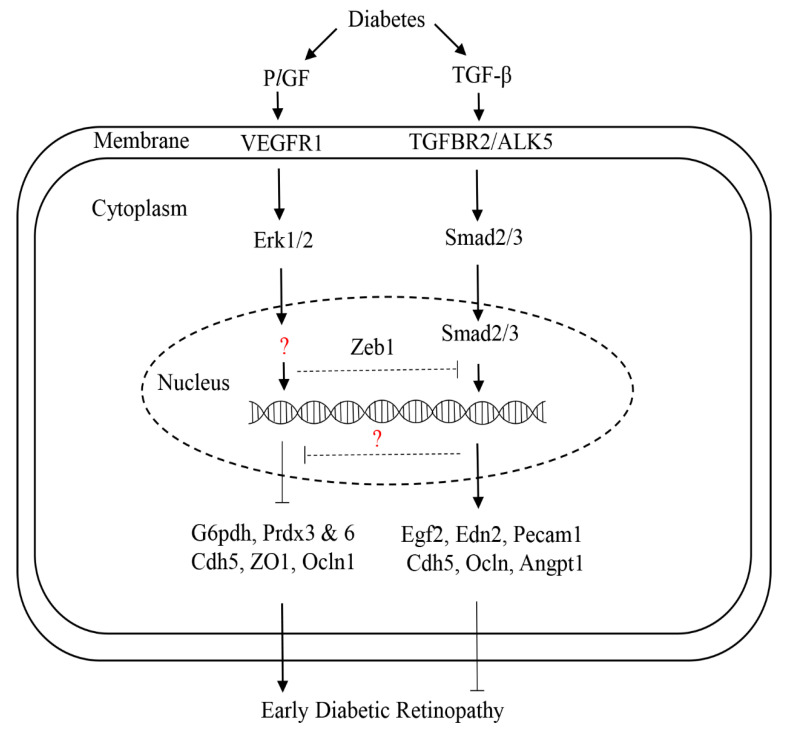
The proposed regulation of P*l*GF and TGF-β in early diabetic retinopathy. Diabetes upregulates both P*l*GF and TGF-β in endothelial cells. P*l*GF can promote early diabetic retinopathy through the activation of VEGFR1 and Erk1/2 signaling, and the expression of downstream target genes, such as G6pdh (pentose phosphate pathway), Prdx3 and 6 (antioxidants), as well as the tight and adhesion junction genes (Cadh5, ZO1, and occludin). The transcription factor(s) that regulate downstream genes’ expressions are to be identified (question maker). Increased TGF-β by diabetes can protect the retina from diabetic injury in the early disease phase through the activation of TGFBR2/ALK5 signaling, which regulates the nuclear translocation of Smad2/3 and then activates the transcription of downstream target genes, such as Egf2, Edn2, and Pcam1. TGF-β can regulate P*l*GF through the unknown factor (question maker). Arrow lines: stimulation. Blunt line: inhibition.

The superfamily of TGF-β contains more than 30 members, including TGF-β1, activin, nodals, and bone morphogenetic proteins (BMP 1-20), and they can bind with several classes of receptors: activin-like kinases (ALKs), TGF-β receptors (TGFBRs), and BMP receptors (BMPRs) [31]. TGF-β signaling plays complex roles in a stage- and context-specific manner during development and disease processes. For instance, the constitutively expressed TGF-β is necessary for the integrity and homeostasis of blood vessels [32]. At the early stage of DR, increased TGF-β plays a protective role in the retinal vasculature. In contrast, it promotes DR progression at the late stage, such as vascular leakage and proliferative DR [31]. The mice with TGFBR2 conditional knockout in ocular tissue (Tgfbr2^Δeye^) presented the features resembling the early pathologies of DR, such as thickening of the basal lamina, vascular leakage, and hemorrhages [33]. Dagher et al. [34] showed that increased TGF-β by diabetes is required for the survival of early diabetic retinal vasculature through ALK5 signaling. In this study, the authors also found that the inhibition of TGF-β signaling by the small ALK5-specific inhibitor SM16 upregulated PGF (or P*l*GF) gene expression, which is in line with the previous studies showing that PGF was the transcriptional target and could be stimulated by TGF- β [35,36]. We recently found that P*l*GF inhibition can activate many gene expressions of the TGF-β signaling pathway, and as such, activating this pathway and likely providing a protective effect on HRECs [37]. Taken together, these findings suggest a negatively regulatory loop between TGF-β and P*l*GF that modulates early DR: TGF-β inhibits the P*l*GF signal, leading to a protective role in the prevention of early DR; conversely, P*l*GF can inhibit the TGF-β signal, causing damaging effects on early pathologies of DR (Figure 3). Furthermore, our bioinformatics approach predicts that the transcription factor zinc finger E-box binding homeobox 1 (ZEB1) may mediate the regulation between the two signaling molecules, which would be interesting to verify experimentally.

### 5.2. Angiopoietin-Tie2 Signaling Pathway

Angiopoietin 1 (Angpt1) is a secreted polypeptide with ~60 kDa molecular weight prominently by pericytes and acts as an agonist of transmembrane receptor Tie2, which is predominantly expressed by endothelial cells. The angpt1-Tie2 signaling axis is indispensable for angiogenesis during embryonic development [38] and plays an essential role in the stability of mature vessels and response to injury [39]. Besides angiogenesis and vascular remodeling, this signaling axis is also involved in vascular leakage and inflammation [40]. On the other hand, VE-PTP and angiopoietin 2 function as the antagonists of Tie2, making them attractive targets for the treatment of angiogenesis-associated disorders such as cancer [41] and diabetic retinopathy [42]. Using a VE-PTP antibody and small inhibitor AKB9778, Shen et al. [43] showed that VE-PTP inhibition stabilizes ocular vasculatures and prevents retinal and choroidal NV induced by hypoxia, laser, and VEGF through activation of the Tie2 signaling pathways, such as Akt, eNOS, and ERK. Despite the EC-specific role, Tie2 can also be expressed by pericytes, and its signaling in pericytes plays a critical role in pericyte migration, endothelial sprouting, postnatal angiogenesis, and tumor angiogenesis [44]. 

### 5.3. JAG1/DDL-Notch Signaling Pathway

During embryonic development, Notch signaling plays an essential role in angiogenesis, vascular cell specification, and A-V vascular patterning [45]. Notch signaling is also expressed and active in the adult vasculature, suggestive of a role in the EC quiescent state and vascular homeostasis. For instance, one study showed that Notch signaling components are expressed by retinal ECs and pericytes, where they modulate cell survival induced by pulsatile flow through the interaction with hedgehog signaling [46]. Another study showed that retinal pericytes express Notch signaling protein and downstream target molecules, which regulate the survival and apoptosis gene expression and promote retinal pericytes survival [47].

More recent studies revealed the critical roles of Notch signaling in vascular function in various pathophysiological conditions. Polacheck et al. [48] demonstrated that Notch 1 regulates shear stress-induced EC barrier function by forming a complex with VE-cadherin and a mechanosensory junctional complex assembly involving LAR, TRIO, and RAC. Miloudi et al. [49] found that the JAG1/DDL4-Notch1 signaling pathway mediates pathological vascular permeability in DR through interaction with VEGFR2 to induce the downstream of Akt/eNOS and Src, and then the VE-cadherin phosphorylation and its dissociation with beta-catenin. Wimmer et al. [50] revealed that Notch signaling is involved in the diabetes-induced basement membrane thickness in a human blood vessel organoids derived from embryonic stem cells (ESCs) or induced pluripotent stem cells (iPSCs). In this study, the authors found that the gamma-secretase inhibitor DAPT inhibits the basement thickening caused by a type 2 diabetic condition (high glucose + TNFα + IL6) in human blood vessel organoids. They also identified the downstream Notch signaling targets such as Jagged 1, DLL1, DLL4, NOCH1, and NOTCH3. The human vascular organoids primarily express NOTCH3 and its downstream target Hes5 pericytes of in nondiabetic and diabetic conditions.

## 6. The Roles of P*l*GF in Angiogenesis and BRB Function

P*l*GF is the second member of the VEGF protein family and a close homolog of the VEGF-A polypeptide. P*l*GF has been characterized as an inflammatory cytokine and also has angiogenic properties, similar to VEGF-A. P*l*GF exerts a substantial effect on blood vessel growth and maturation and has a direct proangiogenic effect on ECs [51]. P*l*GF protein and overexpression can induce DR’s early characteristics and play a potential role in DR progression [52,53,54]. Diabetic human retinas have higher expression of P*l*GF mRNA compared with nondiabetic retinas [55]. In proliferative DR, immunoreactivity for P*l*GF is localized to endothelial and perivascular regions of neovascular membranes [56]. However, P*l*GF’s role in vascular leakage and BRB permeability is not fully understood. To investigate the role of P*l*GF in the BRB function of non-proliferative DR (NPDR), we generated diabetic P*l*GF knockout (Akita.P*l*GF^−/−^) mice by crossing Akita diabetic and P*l*GF^−/−^ mice. Genetic deletion of the P*l*GF gene in mice protects the retina against diabetic damages, such as impaired BRB function. The enhanced expression of BRB-related protein in Akita.PlGF^−/−^ include ZO-1, VE-cadherin, Angiopoietin 1, and Sonic Hedgehog [57]. With the proteomics approach, we have identified a total of 3176 mouse retinal proteins; among them, the expression levels of 107 proteins were significantly different between genetic mutants versus wild-type mice and diabetics versus nondiabetics. Some of these differentially expressed proteins are involved in insulin resistance, antioxidant defense, and neuronal protection, such as Gnb, Prdx6, and Map2 [58].

Furthermore, we performed comparative analyses of transcriptome and proteomics for human retinal ECs (HRECs) and identified differentially expressed genes and proteins in the presence and absence of P*l*GF signaling [59]. Our study further revealed that P*l*GF negatively regulates HRECs barrier function by suppressing the pentose phosphate pathway (PPP) and glucose-6-phosphate dehydrogenase (G6PD) activity, which generates NADPH and reinforces antioxidant defense [60]. Together, our studies have identified the new downstream targets of P*l*GF signaling and give mechanistic insights into the roles of P*l*GF in BRB function relevant to NPDR.

## 7. Multiple Functions of VEGFR1 Signaling

The functions of VEGFR1 are multipotent, depending on the pathophysiological microenvironments, the binding ligands (P*l*GF, VEGF-A, or VEGF-B), and the homo/hetero-dimerization (VEGFR2). VEGFR1 has been reported to play varying roles in vascular development, angiogenesis, cell survival, and inflammation. First of all, VEGFR1 has been characterized as a negative regulator in both embryonic and postnatal vascular development [61,62,63]. Second, VEGFR1 is a positive mediator of pathological angiogenesis in the experimental models of primary tumors and wet age-related macular degeneration (AMD) [64]. Third, VEGFR1 has been reported to promote cell survival under stress conditions. For example, in the oxygen-induced retinopathy (OIR) model, VEGFR1 activation by P*l*GF or TGF-β1 can prevent vessel obliteration or degeneration during the hyperoxia phase, thereby preventing the subsequent vessel proliferation during the hypoxia phase [65,66]. Fourth, VEGFR1 signaling plays a role in regulating the chemotaxis of inflammatory cells [67,68,69]. Fifth, Hagberg et al. [70,71] surprisingly found that VEGFR1 activation by VEGF-B mediates insulin resistance by regulating the uptake of fatty acids in ECs in type 2 diabetes. Sixth, VEGFR1 is expressed by pericytes, where it mediates cell growth and migration [72]. Finally, VEGFR1 also mediates retinal pericyte ablation in an in vivo model of cancer-associated retinopathy [73] and an in vitro BBB model of EC/pericyte cocultures [74], leading to increased vascular permeability in both cases.

Our studies have shown VEGFR1’s roles in ischemic and inflammatory retinopathies using the experimental models of retinopathy of prematurity (ROP), age-related macular degeneration (AMD), and DR [64,75,76,77]. We have found that the VEGFR1 blockade prevents BRB breakdown, retinal neovascularization (NV), and other complications of DR [64,75,77] and that the potency of anti-VEGFR1 is comparable to that of anti-VEGF or anti-VEGFR2 [64]. We have also found that VEGFR1 is a crucial receptor for microglia or macrophage activation in response to laser injury [75].

## 8. Pericytes Regulation of ECs Integrity and Function

Pericytes maintain vascular stability and enhance EC barrier function by direct contact and paracrine regulation. As discussed above, pericytes can secrete and activate the signaling molecules that regulate PC-EC crosstalk and EC function, such as ANGPT1 and TGF-beta. Endothelial proliferative sprouting in angiogenic blood vessel growth is critical to establish a functional vascular capillary plexus during development and vascular remodeling. To investigate the regulatory role of pericytes in these processes, Eilken et al. [78] generated the diphtheria toxin/DTR-mediated pericytes depletion mouse model (DTR^iPC^ double transgenic mice). They showed that pericytes regulate endothelial proliferation and sprouting in retinal angiogenesis through the VEGF(A)-VEGFR2 signaling pathway. The authors also generated another transgenic mouse line (Flt1iPC), in which VEGFR1 was inactivated specifically in pericytes. They found that these mice photocopied the key phenotypes observed for those pericytes-depleted mice regarding the defects of endothelial sprouting and filopodia formation. Therefore, the conclusion was drawn about the paracrine regulation of VEGF-dependent ECs spouting through pericytes VEGFR1 signaling.

To study the role of pericytes in the formation, maturation, and maintenance of the blood-retinal barrier, Park et al. [79] depleted PDGFB specifically in ECs by generating a transgenic mouse line (Pdgfb^iΔEC^), which had reduced pericytes coverage. In this mouse line, the authors observed that defective pericytes coverage results in BRB impairment and increased leakage in developing retinal vasculatures, which impairs vision function. The activation of FOXO1 and ANGPT2 contributes to pathological events, which could be alleviated by these molecular activities’ inactivation. In contrast, ANGPT1 and Tie2 were shown to be critical for retinal vascular growth and BRB maturation. Surprisingly, the authors found that pericytes are not the primary source of ANGPT1, but expressed by other cell types, such as retinal neurons. This new finding challenges the previous reports that pericytes mainly express ANGPT1 and contribute to vessel stabilization [39]. Given that pericytes are highly heterogeneous, the clinical relevance of these new findings in retinal vascular disorders such as DR is to be further investigated, as commented by Santos et al. [80].

## 9. Exosome Regulation of EC Integrity and Functions

Extracellular exosomes encompassing proteins, lipids, and RNA (e.g., miRNA) have been increasingly recognized to regulate cell-to-cell communication between ECs and pericytes. The pericytes activated by the hypoxia-inducible factor (HIF) pathway in a hypoxia condition can secrete exosomes that regulate EC migration, sprouting, and angiogenesis in the wound healing model and spinal cord explant cultures [81]. Pericyte exosomes can improve microcirculation and protect the blood-spinal cord barrier from spinal cord injury in mice, which is related to increased HIF-1α, Bax, aquaporin-4, and MMP2, as well as decreased claudin-5 and Bcl-2 [82]. Besides pericytes, exosomes from neurons, glia, and endothelial cells, as well as the circulation, can regulate EC integrity and intercellular crosstalk within NVU in both physiological and pathological conditions [83,84,85].

More recently, Liu et al. [86] identified a new circular RNA named cPWWP2A that is upregulated in the db/db diabetic mouse retina and has a human homologous gene. The authors first showed cPWWP2A silencing mediated by AAV-shRNA, which aggravates diabetes-induced retinal microvascular injuries and dysfunctions, such as pericytes loss and microaneurysms acellular capillary, vascular leakage, and increased inflammation. Conversely, cPWWP2A overexpression alleviates these diabetic complications. The authors then found that cPWWP2A regulates pericyte function, pericyte-EC crosstalk, and vascular dysfunction by acting as a sponge of miR-579 and targeting Angiopoietin 1, occluding, and SIRT1 both in vitro and in vivo. Lastly, the authors uncovered that cPWWP2A is produced by pericytes and transported to ECs through exosomes [86]. This study is interesting because it demonstrates a new regulatory mechanism of EC-PC crosstalks that can be potentially used as therapeutic innervations.

## 10. Pericyte-EC Communication Impairments in the Retinal Vascular Diseases

Dysregulation of pericyte-EC communication occurs in many retinal vascular diseases, which in reverse aggravates the disease progression, such as ROP, DR, retinal vein occlusion (RVO), and Norrie disease. Loss of pericytes and ECs, stressing environmental conditions, and aberrant signaling pathways can cause pericyte-EC miscommunication in retinal vascular diseases. For example, in the OIR models and the ROP infants, the obliteration of developing vasculature caused by escalated oxygen levels results in retinal ischemia followed by pathological angiogenesis [87]. Inflammation and oxidative stress can drive vascular damage, resulting in increased BRB permeability in ischemia and DR [3,4]. Norrin/Frizzled4 signaling is perturbed in Norrie disease, leading to abnormal vasculatures and BRB/BBB leakage in the brain and the retina [26,88]. Glucocorticoids and various anti-VEGF agents, such as Avastin, Lucentis, and Aflibercept, have been exploited to treat patients with leaky retinal blood vessels and pathological angiogenesis concerning diabetic macular edema and proliferative diabetic retinopathy [2,89,90]. Cell-based therapies, such as ESCs, iPSCs, and progenitor cells, hold the promise for replacing degenerative vascular cells [91].

## 11. Experimental Models to Study Pericyte-Endothelial Interactions

**Figure 4:**
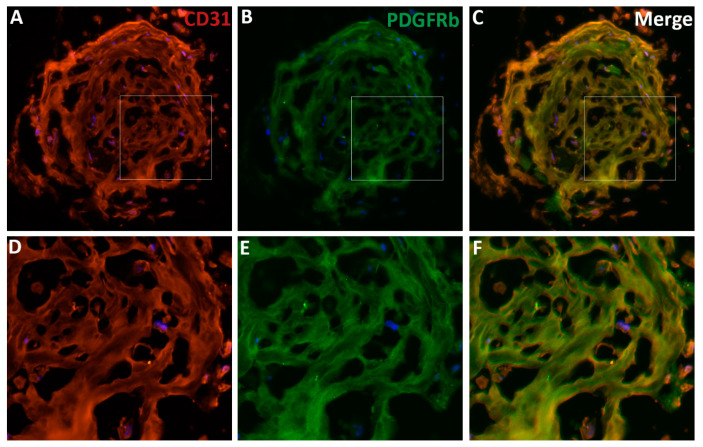
The features of vascular organoids derived from human-induced pluripotent stem cells. (**A**–**C**) The vascular organoids are differentiated from human induced pluripotent stem cells (iPSCs). The 10-micron cryosections are prepared and stained with the endothelial cell (ECs) marker CD31 (or PECAM1, **A**, red) and the pericyte marker PDGFRb (**B**, green). The nuclei were stained with DAPI (blue). (**C**) The merged image shows the co-localization of the two markers in the blood vessels. (**D**–**E**) the enlarged images of the boxed areas in panels **A**–**C**. Note that the integral vascular networks of organoids vasculatures with vessel lumens, ECs, and pericytes in the blood vessels. Human organoids can be an excellent model to study EC-pericyte interaction and other human vascular diseases’ pathophysiology.

**Figure 5:**
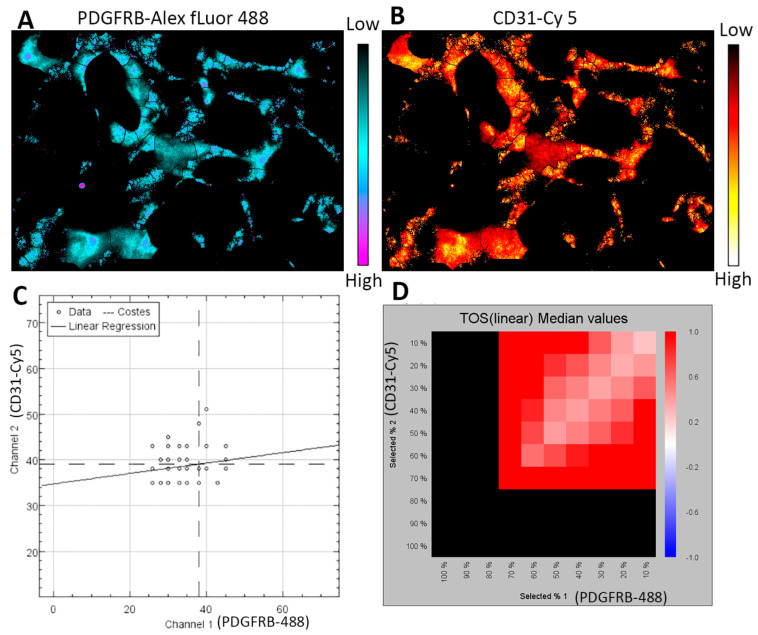
Co-localization analysis of pericytes and endothelial cells in the organoid vasculature. Part of the organoid vasculature (1/6) is used for analysis with the ImageJ software and EzColocalization plugin [96]. (**A**,**B**) The heat maps show the localization of PDGFRB and CD31 staining signals in the vessels. The scale bars indicate the signal intensity from low to high. (**C**) The scatterplot shows the relationship between the signal intensity for PDGFRB-Alex fluor 488 and CD31-Cy 5 channels. Note that (1) the numbers of data represent the vessel segments identified by the software, and (2) data with similar signal intensity for each channel indicate colocalization versus anti-colocalization by the differences in signal intensity. (**D**) The metric matrix for the threshold overlap score (TOS) linear median values. The X-axis and Y-axis values are the top percentile (F_T_) of threshold pixels for signal intensity. The black color is not informative. The red color indicates co-localization.

ECs and pericytes can be co-cultured to study their interactions in vitro. D’Amore et al. [92,93] used primary ECs and pericytes from the bovine retina to elucidate their roles in vessel development and function, as well as the regulatory mechanisms. We have co-cultured primary ECs and pericytes from the human retina to investigate the regulation of HRECs-HRPCs interaction by P*l*GF signaling (not yet published). Many transgenic mouse models have been created to trace pericytes in the developing and mature vasculatures, such as the inducible models of PDGFRb-CreER^T2^ and tbx18-CreER^T2^ transgenic mice (see review [11]). Additionally, animal models have been created to interrogate the pathological mechanisms regulating pericyte-EC miscommunication under stress and disease conditions, such as OIR and diabetic animals (see review [94]). Wimmer et al. [50] recently created three-dimensional (3D) blood vessel organoids derived from ESCs and iPSCs; this 3D culture system provides a new attractive model because the vascular networks have many features similar to human microvasculatures in terms of components, architectures, and functions. By using the culture protocols described by the authors [95], we have successfully cultured the 3D vascular organoids from human iPSCs to study the pericytes-endothelial interactions and the regulatory mechanisms. The vascular networks in our organoids are indeed integral. They include the comprehensive features of human vasculatures, such as CD31^+^ ECs forming blood lumens and PDGFbR^+^ pericytes properly covering the ECs on the blood vessels, which together form vasculature networks in the organoids (Figure 4). The EC-pericyte close associations in organoid vasculature are demonstrated by co-localization analysis with the ImageJ and EzColocalization plugin (Figure 5) [96].

## 12. Conclusions and Prospects

Retinal microvasculature is a window to the brain microvasculature, and both microvascular systems possess blood-barrier properties and form NVU with neurons and glial cells. Pericyte-endothelial interactions are fundamental for NVU function and vascular homeostasis. Many essential signaling pathways have been characterized for the modulation of pericyte-endothelial interactions, which are disrupted in many CNS vascular disorders. How the altered signaling pathways and pathological conditions cause vascular abnormalities and disease phenotypes is not entirely understood. Advancements in the knowledge about pericyte-EC interaction and communication can facilitate the design of the preventative and interventional therapeutics for retinal vascular diseases. Besides traditional two-dimensional (2D) cell cultures and animal models, 3D blood vessel organoids derived from human ESCs and iPSCs provide a new model system to interrogate organogenesis mechanisms to understand disease etiology and develop precision or personalized medicines for vascular disorders.

## Abbreviation

3D	three-dimensional
α-SMA	alpha-smooth muscle actin
ALKs	activin-like kinases
AMD	age-related macular degeneration
Angpt1	Angiopoietin 1
A-V	arterial-venous
BBB	blood-brain barrier
BM	bone marrow
BMP	bone morphogenetic proteins
BMPRs	BMP receptors
BRB	blood-retinal barrier
CTGF	connective tissue growth factor
CNS	central nervous system
DR	diabetic retinopathy
ECM	extracellular matrix
ECs	endothelial cells
ESCs	embryonic stem cells
FGF	fibroblast growth factor
G6PD	glucose-6-phosphate dehydrogenase
HIF	hypoxia-inducible factor
HRECs	human retinal endothelial cells
IPL	inner plexiform layer
iPSCs	induced pluripotent stem cells
NFL	nerve fiber layer
NVU	neurovascular unit
OPL	outer plexiform layer
OIR	oxygen induced retinopathy
PCs	pericytes
PDGFB	palate-derived growth factor subunit B
PDGFR-β	platelet-derived growth factor receptor beta
PFKFB3	6-Phosphofructo-2-Kinase/Fructose-2,6-Biphosphatase 3
P*l*GF or PGF	placental growth factor
PPP	pentose phosphate pathway
ROP	retinopathy of prematurity
RVO	retinal vein occlusion
S1P	Sphingosine-1-phosphage
Sem3A	semaphoring3A
TGF-β	transforming growth factor-beta
TGFBRs	TGF-β receptors
VSMCs	vascular smooth muscle cell
VEGF	vascular endothelial growth factor
ZEB1	zinc finger E-box binding homeobox 1
